# Supplementary material for: A third-generation dispersion and third-generation hydrogen bonding corrected PM6 method: PM6-D3H+
Source: PeerJ. 2014 Jun 19;2:e449. doi: 10.7717/peerj.449 (PMC4081274; doi:10.7717/peerj.449)
Supplement: Supplemental Information 1 [file peerj-02-449-s001.pdf]

Supplementary Information

- S.1 S22 and S66 Complexes optimized with OPTTOL = 0.0001
- S.2 S22 and S66 Complexes with imaginary frequencies
- S.3 GAMESS Header examples

S.1 S22 and S66 Complexes optimized with OPTTOL = 0.0001

Table S1

Set	ID	Name
s22	08	Methanedimer
s22	12	Pyrazinedimer
s22	18	Benzeneammoniacomplex
s66	04	WaterPeptide
s66	23	AcNH2Uracil[a]
s66	33	PyridineEthene
s66	37	CyclopentaneNeopentane
s66	38	CyclopentaneCyclopentane
s66	57	BenzenePeptideNHpi
s66	65	PyridineEthyne[a]
s66	66	MeNH2Pyridine

[a] Necessary to also set ihrep to 20.

S.2 S22 and S66 Complexes with imaginary frequencies

Table S2

Set	ID	Name	No. i-freq
		PM6[a]	
s66	30	BenzeneEthene	1
s66	65	PyridineEthyne	1
		PM6-D3H+[a]	
s22	01	Ammoniadimer	1
s22	15	Adeninethyminecomplexstack	1
s22	20	BenzenedimerTshaped	1
s66	16	PeptideWater	1
s66	30	BenzeneEthene	1
s66	42	UracilCyclopentane	1

[a] The calculations have been done using the GAMESS software.

[b] The calculations have been done using the MOPAC software.

Table S3

Set	ID	Name	No. *i*-freq
		PM6[b]	
s22	01	Ammoniadimer	2
s22	04	Formamidedimer	2
s22	05	Uracildimerhbonded	2
s22	06	2pyridoxine2aminopyridinecomplex	3
s22	07	AdeninethymineWatsonCrickcomplex	2
s22	11	Benzenedimerparalleldisplaced	1
s22	14	Indolebenzenecomplexstack	1
s22	19	BenzeneHCNcomplex	2
s22	20	BenzenedimerTshaped	3
s66	04	WaterPeptide	1
s66	05	MeOHMeOH	1
s66	06	MeOHMeNH2	1
s66	08	MeOHWater	1
s66	09	MeNH2MeOH	1
s66	10	MeNH2MeNH2	1
s66	14	PeptideMeNH2	2
s66	16	PeptideWater	1
s66	17	UracilUracilBP	2
s66	18	WaterPyridine	1
s66	20	AcOHAcOH	1
s66	22	AcOHUracil	1
s66	23	AcNH2Uracil	2
s66	25	PyridinePyridinepipi	1
s66	28	BenzeneUracilpipi	1
s66	35	NeopentanePentane	1
s66	36	NeopentaneNeopentane	6
s66	37	CyclopentaneNeopentane	1
s66	39	BenzeneCyclopentane	1
s66	41	UracilPentane	1
s66	42	UracilCyclopentane	2
s66	44	EthenePentane	1
s66	47	BenzeneBenzeneTS	2
s66	48	PyridinePyridineTS	1
s66	49	BenzenePyridineTS	1
s66	59	EthyneWaterCHO	2
s66	63	BenzeneAcOH	1
s66	66	MeNH2Pyridine	3

[a] The calculations have been done using the GAMESS software.

[b] The calculations have been done using the MOPAC software.

Table S4

Set	ID	Name	No. i-freq
		PM6-DH+[b]	
s22	01	Ammoniadimer	2
s22	05	Uracildimerhbonded	1
s22	06	2pyridoxine2aminopyridinecomplex	2
s22	07	AdeninethymineWatsonCrickcomplex	1
s22	10	BenzeneMethanecomplex	3
s22	18	Benzeneammoniacomplex	2
s22	19	BenzeneHCNcomplex	2
s22	20	BenzenedimerTshaped	2
s22	21	IndolebenzeneTshapecomplex	1
s66	08	MeOHWater	2
s66	10	MeNH2MeNH2	1
s66	12	MeNH2Water	1
s66	13	PeptideMeOH	1
s66	14	PeptideMeNH2	1
s66	15	PeptidePeptide	1
s66	16	PeptideWater	2
s66	17	UracilUracilBP	2
s66	19	MeOHPyridine	1
s66	20	AcOHAcOH	2
s66	22	AcOHUracil	1
s66	23	AcNH2Uracil	2
s66	24	BenzeneBenzenepipi	1
s66	25	PyridinePyridinepipi	2
s66	36	NeopentaneNeopentane	9
s66	42	UracilCyclopentane	2
s66	45	EthynePentane	1
s66	46	PeptidePentane	1
s66	47	BenzeneBenzeneTS	1
s66	48	PyridinePyridineTS	1
s66	49	BenzenePyridineTS	2
s66	52	BenzeneAcOHOHpi	2
s66	55	BenzeneMeOHOHpi	1
s66	66	MeNH2Pyridine	1

[a] The calculations have been done using the GAMESS software.
[b] The calculations have been done using the MOPAC software.

S.3 GAMESS Header examples

PM6-D3H+ Optimization and vibrational analysis

```
$basis
   gbasis=PM6-D3H+    ! Use the PM6 method w/ D3 and H+ correction
$end

$contrl
   scftyp=RHF         ! Use Restricted Hartree-fock
   icharg=0           ! Total molecule charge
   runtyp=optimize    ! Do a geometry optimization
$end

$scf
   npunch=1           ! less output during SCF iterations
$end

$statpt
   opttol=5.0e-4      ! convergence critria
   nstep=500          ! Maximum no. of steps

   hssend=.T.         ! do hessian calculation after optimization
$end

$force
   nvib=2             ! force calculation using centered finite difference scheme
   method=seminum     ! Use semi-numerical scheme for force calculation
$end
```

PM6-D3H+/PCM Optimization and vibrational analysis

```
$basis
   gbasis=PM6-D3H+    ! Use the PM6 method w/ D3 and H+ correction
$end

$contrl
   scftyp=RHF         ! Use Restricted Hartree-fock
   icharg=0           ! Total molecule charge
   runtyp=optimize    ! Do a geometry optimization
$end

$scf
   npunch=1           ! less output during SCF iterations
$end

$statpt
   opttol=5.0e-4      ! convergence critria
   nstep=500          ! Maximum no. of steps

   hssend=.T.         ! do hessian calculation after optimization
$end

$force
   nvib=2             ! force calculation using centered finite difference scheme
   method=seminum     ! Use semi-numerical scheme for force calculation
$end

! Solvent settings
$pcm
   solvnt=WATER
   mxts=15000         ! The maximum number of tesserae
$end

$tescav
   mthall=4           ! Use the FIXPVA scheme
   ntsall=60          ! The density of tesserae
$end
```

PM6-D3H+/PCM Optimization GAMESS header w/ convergence help

```
$basis
   gbasis=PM6-D3H+    ! Use the PM6 method w/ D3 and H+ correction
$end

$contrl
   scftyp=RHF         ! Use Restricted Hartree-fock
   icharg=0           ! Total molecule charge
   runtyp=optimize    ! Do a geometry optimization
$end

$scf
   npunch=1           ! less output during SCF iterations
$end

$statpt
   opttol=1.0e-4      ! convergence critria
   nstep=500          ! Maximum no. of steps

   hssend=.T.         ! do hessian calculation after optimization

   ihrep=20           ! Update Hessian every nth step
   projct=.F.         ! flag to eliminate translation and rotational degress of freedom
$end

$force
   nvib=2             ! force calculation using centered finite difference scheme
   method=seminum     ! Use semi-numerical scheme for force calculation
$end

! Solvent settings
$pcm
   solvnt=WATER
   mxts=15000         ! The maximum number of tesserae
$end

$tescav
   mthall=4           ! Use the FIXPVA scheme
   ntsall=60          ! The density of tesserae
$end
```

S.4 Selected Complexes from JSCH-2005

Table S5

BEGDB ID	Name
1018	G...U wobble
1017	I...C WC
1020	U...U
1021	U...U pl
1084	A...T S1
1014	A...T WC
1082	G...C S
1012	G...C WC(1)
1015	mA...mT H
1085	mA...mT S
1083	mG...mC S
1013	mG...mC WC
